# Signal Processing in Periodically Forced Gradient Frequency Neural Networks

**DOI:** 10.3389/fncom.2015.00152

**Published:** 2015-12-24

**Authors:** Ji Chul Kim, Edward W. Large

**Affiliations:** Department of Psychological Sciences, University of ConnecticutStorrs, CT, USA

**Keywords:** non-linear oscillation, neural networks, synchronization, signal processing, auditory perception

## Abstract

Oscillatory instability at the Hopf bifurcation is a dynamical phenomenon that has been suggested to characterize active non-linear processes observed in the auditory system. Networks of oscillators poised near Hopf bifurcation points and tuned to tonotopically distributed frequencies have been used as models of auditory processing at various levels, but systematic investigation of the dynamical properties of such oscillatory networks is still lacking. Here we provide a dynamical systems analysis of a canonical model for gradient frequency neural networks driven by a periodic signal. We use linear stability analysis to identify various driven behaviors of canonical oscillators for all possible ranges of model and forcing parameters. The analysis shows that canonical oscillators exhibit qualitatively different sets of driven states and transitions for different regimes of model parameters. We classify the parameter regimes into four main categories based on their distinct signal processing capabilities. This analysis will lead to deeper understanding of the diverse behaviors of neural systems under periodic forcing and can inform the design of oscillatory network models of auditory signal processing.

## 1. Introduction

Neural oscillation is observed throughout the central nervous system and has been suggested to have important functional roles in peripheral, subcortical, and cortical processing (Buzsáki and Draguhn, 2004; Sejnowski and Paulsen, 2006; Koepsell et al., 2010). In the auditory system, oscillatory activities are found at all levels of the pathway, including spontaneous oscillation of haircell bundles (Crawford and Fettiplace, 1985; Martin et al., 2003; Ramunno-Johnson et al., 2009), cochlear dynamics poised near a critical point of oscillatory instability called the Hopf bifurcation (Camalet et al., 2000; Ospeck et al., 2001), chopper and onset cells in the cochlear nucleus mode-locking to periodic stimulation (Laudanski et al., 2010), and the inferior colliculus neurons spontaneously firing at audible frequencies (Schwarz et al., 1993). Mathematical models of non-linear oscillation are used to explain the oscillatory response of these auditory areas to periodic stimulation (Eguíluz et al., 2000; Jülicher et al., 2001; Meddis and O'Mard, 2006; Laudanski et al., 2010; Fredrickson-Hemsing et al., 2012). Forced non-linear oscillator models share important behaviors including synchronization and non-linear compression, but at the same time they exhibit diverse dynamical responses to periodic signals.

To apprehend the full range of possible behaviors, it is important to understand how the dynamical properties of forced non-linear oscillations vary from one parameter regime to another. Here we provide a mathematical analysis of an oscillatory network model that is widely used in auditory modeling. We enumerate the full set of behaviors the model exhibits under periodic forcing. This analysis reveals the signal processing capabilities of a large class of dynamical systems.

Mathematical models of individual neurons and neural populations vary in their degree of physiological detail and mathematical complexity. Biophysically detailed models describe neurophysiological mechanisms with variables representing physical or chemical quantities and can exhibit a range of diverse behaviors observed in the original biological system (e.g., the Hodgkin–Huxley model; Hodgkin and Huxley, 1952). Other models take simpler mathematical forms and capture the local dynamics of a select behavior with fewer variables, thus making the essential dynamics more transparent and amenable to mathematical analysis (Hoppensteadt and Izhikevich, 2001). The canonical model for gradient frequency neural networks (abbr. GrFNNs) is one such simple mathematical model that describes the dynamical properties shared by networks of oscillatory neural populations tuned to a gradient of distinct frequencies, which are found at various stages of the auditory system (Large et al., 2010). The model assumes that each oscillatory neural population (or neural oscillator) in the network is poised near a Hopf bifurcation point, which is a transition between quiescence and spontaneous oscillation (Guckenheimer and Holmes, 1983). The canonical model for gradient frequency networks can be considered as an extension of the canonical model for homogeneous (equal or very close) frequency networks of neural oscillators (Hoppensteadt and Izhikevich, 1997) into multi-frequency systems.

When the oscillators are tuned to logarithmically spaced frequencies, the dynamics of the canonical model for gradient frequency neural networks (Large et al., 2010) is described by

(1) $$\begin{array}{r} \tau_{i}{ż}_{i}=z_{i}\left(\alpha +\text{i}2\pi +\left(\beta_{1}+\text{i}\delta_{1}\right)|z_{i}{|}^{2}+\frac{\epsilon \left(\beta_{2}+\text{i}\delta_{2}\right)|z_{i}{|}^{4}}{1-\epsilon |z_{i}{|}^{2}}\right)+RT, \end{array}$$

where *z*_*i*_ is a complex state variable representing the amplitude and phase of synchronized firing of the *i*th neural population in the network, ϵ is a small real number indicating the degree of non-linearity in the network, a dot over a variable denotes its time derivative, and the roman i denotes the imaginary unit. The right-hand side of Equation (1) consists of the intrinsic terms (all right-hand-side terms except *RT*), which determine the autonomous behavior of the model, and the input terms (*RT*), which describe the interaction of the model with the input. The bandwidth of oscillators is constant in logarithmic frequency because the equation is scaled by the time constant τ_*i*_, which is the reciprocal of the natural frequency *f*_*i*_. The intrinsic parameters α, β_1_, and β_2_ control the bifurcation of autonomous behavior (see below), and δ_1_ and δ_2_ determine the dependency of autonomous frequency on amplitude. *RT* (resonant terms) is a sum of input terms that are potentially resonant to the oscillator's dynamics, which could include both linear and non-linear terms involving external forcing and/or coupling with other oscillators in the network (see Large et al., 2010; Lerud et al., 2014, for possible closed-form expressions of *RT*). Commonly, models of oscillation near a Hopf bifurcation have intrinsic terms only up to the third order [i.e., the cubic term with β_1_ and δ_1_ in Equation (1); see Eguíluz et al., 2000; Jülicher et al., 2001 for instance], but the canonical model retains a full series of higher-order terms expressed as a geometric sum (i.e., the term with β_2_ and δ_2_) to cope with the high-order non-linear input terms in *RT*.

With the high-order terms governing non-linear interactions of an oscillator with the external signal and also with other oscillators in the network, the canonical model captures the general properties of non-linear dynamics arising in gradient frequency oscillator networks. When driven by an external signal, the oscillators in the canonical model produce non-linear responses containing not only the frequencies in the signal but also non-linear combinations of their natural frequencies and the signal frequencies. Non-linear coupling in the network transforms the signal further by introducing frequencies arising from resonance between oscillators tuned to different frequencies. As a generic model of non-linear, multi-frequency transformation of acoustic signals into neural firing patterns occurring in the auditory system, the canonical model has been used to model auditory processing and music perception. Multi-layer gradient frequency networks were used to model cochlear dynamics by fitting the auditory nerve tuning curves of macaque monkeys (Lerud et al., 2015) and to model the human brainstem frequency-following response to musical intervals by fitting the spectra of auditory evoked potentials (Large and Almonte, 2012; Lerud et al., 2014). Also, both model simulations and analytic predictions were used to explain the perception of musical tonality (Large, 2010; Large et al., in press) and the beat perception in musical rhythm (Large et al., 2015).

Despite its simple mathematical form, the canonical model for gradient frequency neural networks is still difficult to analyze in its entirety because its dynamics is determined by complex interactions among multiple network components. Oscillators in the network are driven by external forcing and at the same time receive input from other oscillators, and both types of interaction may involve linear and/or non-linear coupling which can evolve over time via a generalized form of Hebbian plasticity (Hoppensteadt and Izhikevich, 1996; Large, 2011). Our approach is to analyze individual components of the network separately and attempt to understand its overall dynamics as a combination of its component dynamics. In this paper, we set to analyze and categorize the driven behaviors of canonical oscillators under periodic forcing.

## 2. Methods

We consider the following differential equation describing an oscillator in the canonical model (or simply, a canonical oscillator) driven by sinusoidal forcing of fixed frequency, ω_0_, and amplitude, *F*:

(2) $$\begin{array}{r} ż=z\left(\alpha +\text{i}\omega +\beta_{1}|z{|}^{2}+\frac{\epsilon \beta_{2}|z{|}^{4}}{1-\epsilon |z{|}^{2}}\right)+Fe^{\text{i}\omega_{0}t}, \end{array}$$

where ω = 2π*f* is the radian natural frequency. To understand the response of a gradient frequency network, our analysis will focus on how the driven state of an oscillator changes as a function of its natural frequency. Since only one oscillator is analyzed, the subscript *i* in Equation (1) is dropped and the scaling factor is ignored (but see Section 3.6 for frequency scaling of log frequency networks). For the simplicity of analysis, the δ parameters are set to zero, meaning that the intrinsic frequency of the oscillator is not dependent on its amplitude.

The autonomous behavior of the oscillator (i.e., when *F* = 0) is readily seen when it is brought to polar coordinates using *z* = *re*^iϕ^. Then, the amplitude and phase dynamics are described by

$$\left\{ \begin{array}{l} \dot{r}=\alpha r+\beta_{1}r^{3}+\frac{\epsilon \beta_{2}r^{5}}{1-\epsilon r^{2}}\\ \dot{\phi }=\omega . \end{array}\right.$$

**Figure 1:**
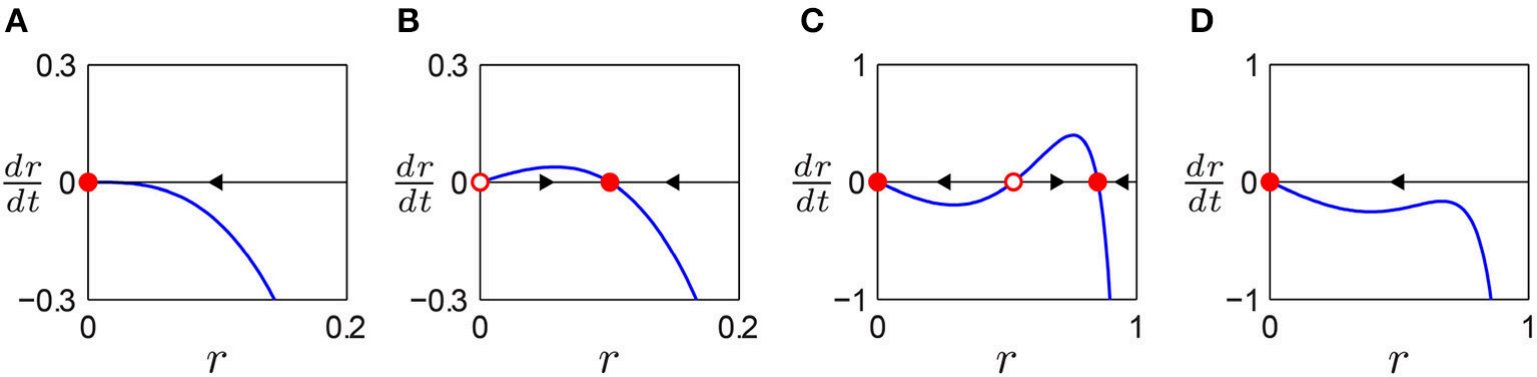
**Autonomous behavior of a canonical oscillator in different parameter regimes**. Amplitude vector field is shown for **(A)** a critical Hopf regime (α = 0, β_1_ < 0, β_2_ = 0), **(B)** a supercritical Hopf regime (α > 0, β_1_ < 0, β_2_ = 0), **(C)** a supercritical double limit cycle regime (α < 0, β_1_ > 0, β_2_ < 0, local max > 0), and **(D)** a subcritical double limit cycle regime (α < 0, β_1_ > 0, β_2_ < 0, local max < 0). Filled circles indicate stable fixed points (attractors) and empty circles unstable fixed points (repellers). Arrows indicate the direction of trajectories in the vector field.

The first equation above defines the amplitude vector field, which shows whether the amplitude increases, decreases, or is stationary over time at a given amplitude value (Figure 1). A fixed point in the vector field, which is obtained by solving *ṙ* = 0, represents a steady-state amplitude of the autonomous oscillator. The stability of a fixed point determines if it is an attractor, to which the oscillator returns after small perturbation, or a repeller, from which the oscillator diverges when perturbed. The second equation above shows that the phase ϕ advances at the constant rate of ω.

Depending on the values of α, β_1_, and β_2_, the autonomous amplitude vector field can have one of four distinct topologies. When *ṙ* decreases monotonically as *r* increases, the origin is the only fixed point which is stable as the arrow indicates (Figure 1A). An oscillator with this type of amplitude vector field decays to zero while oscillating at its natural frequency. A representative parameter regime for this type is the critical point of a supercritical Hopf bifurcation (α = 0, β_1_ < 0). (A subcritical Hopf bifurcation occurs when α = 0 and β_1_ > 0). When *ṙ* increases from the origin and then decreases after a local maximum, there is a stable non-zero fixed point while the origin is rendered unstable (Figure 1B). An oscillator of this type shows spontaneous oscillation at the amplitude of the stable fixed point (unless the initial condition is zero). The supercritical branch of a supercritical Hopf bifurcation (α > 0, β_1_ < 0) is an example. When there are three fixed points with two local extrema, two of the fixed points are stable, indicating bistability between equilibrium at zero and spontaneous oscillation at a non-zero amplitude (Figure 1C). As the local maximum in the vector field moves below the *r* axis by, say, decreasing β_1_, the two non-zero fixed points collide and vanish (Figure 1D). This transition is called a double limit cycle (hereafter, DLC) bifurcation since it involves two limit cycles (closed orbits) in the (*r*, ϕ) plane, one stable and the other unstable. Thus, we call the regime shown in Figure 1C (α < 0, β_1_ > 0, β_2_ < 0, local max > 0) *supercritical DLC* and the one shown in Figure 1D (α < 0, β_1_ > 0, β_2_ < 0, local max < 0) *subcritical DLC*. The subcritical DLC regime has only one stable fixed point at zero but is different from the critical Hopf regime (Figure 1A) in that it has a local maximum in the vector field. We will show that a subcritical DLC oscillator has different sets of driven behaviors from a critical Hopf oscillator despite their qualitatively identical autonomous behavior.

**Figure 2:**
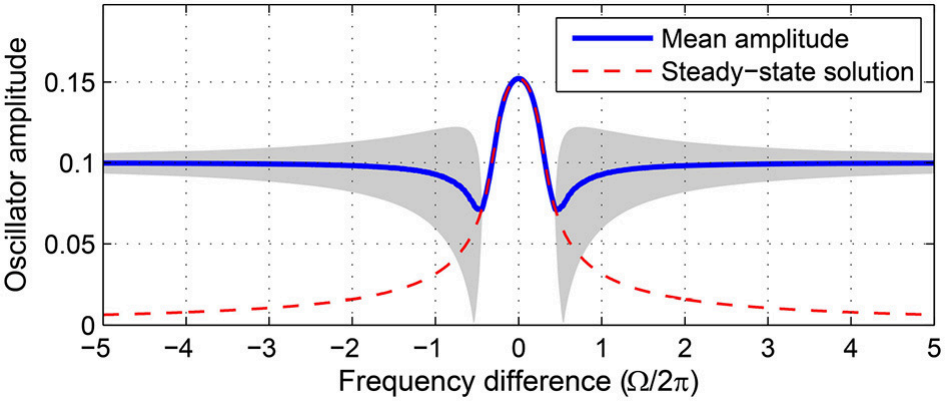
**Not all steady-state solutions are stable**. Time-averaged amplitude of a canonical oscillator driven by a sinusoidal input obtained from numerical simulations (solid line) is compared with the steady-state solution of Equation 3 (dashed line). Steady-state solutions are stable attractors where they match the mean amplitudes from simulations. The gray shade indicates the range of amplitude fluctuation where the oscillator does not stably phase-lock to the input. Compare with the stability analysis of the same oscillator (α = 1, β_1_ = −100, β_2_ = 0, *F* = 0.2) shown in **Figure 6**.

To examine how a canonical oscillator responds to external forcing, we bring Equation (2) to polar coordinates, again using *z* = *re*^iϕ^, and express its dynamics in terms of the relative phase ψ = ϕ − ω_0_*t* so that a stable fixed point in (*r*, ψ) indicates a phase-locked state:

(3) $$\left\{ \begin{array}{l} \dot{r}=\alpha r+\beta_{1}r^{3}+\frac{\epsilon \beta_{2}r^{5}}{1-\epsilon r^{2}}+F\cos\psi \\ \dot{\psi }=\Omega -\frac{F}{r}\sin\psi , \end{array}\right.$$

where Ω = ω − ω_0_ is the frequency difference between the oscillator and the input. We evaluate the stability of fixed point(s) for a range of forcing parameters Ω and *F* wide enough to encompass all possible qualitatively different driven behaviors of the four regimes of intrinsic parameters introduced above. Stability analysis is crucial for understanding the dynamical responses of the driven oscillator because not all fixed points are stable. The existence of a steady-state solution (i.e., a fixed point) does not guarantee that the oscillator phase-locks to the forcing, since the fixed point could be unstable (Figure 2).

Fixed points, notated as (r^*^, ψ^*^), are obtained by solving the steady-state equations *ṙ* = 0 and $\dot{\psi }=0$ simultaneously, and the stability of each fixed point is determined by evaluating the Jacobian matrix, *J*, at the fixed point:

$$\begin{array}{l} J={\left(\begin{array}{cc} \frac{\partial p}{\partial r} & \frac{\partial p}{\partial \psi }\\ \frac{\partial q}{\partial r} & \frac{\partial q}{\partial \psi } \end{array}\right)}_{\left(r^{*},\psi^{*}\right)}\\ =\left(\begin{array}{cc} \alpha +3\beta_{1}r^{*2}+\frac{\epsilon \beta_{2}r^{*4}(5-3\epsilon r^{*2})}{{\left(1-\epsilon r^{*2}\right)}^{2}} & -F\sin\psi^{*}\\ 2\delta_{1}r^{*}+\frac{2\epsilon \delta_{2}r^{*3}(2-\epsilon r^{*2})}{{\left(1-\epsilon r^{*2}\right)}^{2}}+\frac{F}{r^{*2}}\sin\psi^{*} & -\frac{F}{r^{*}}\cos\psi^{*} \end{array}\right), \end{array}$$

where *ṙ* = *p*(*r*, ψ, ⋯) and $\dot{\psi }=q\left(r,\psi ,\cdots \;\right)$ in Equation (3). Let T and Δ be the trace and determinant of the Jacobian matrix. Then its eigenvalues are $\lambda_{1,2}=\frac{1}{2}\left(\text{T}\pm \sqrt{{\text{T}}^{2}-4\Delta }\right)$, and local trajectories near the fixed point have the form $c_{1}e^{\lambda_{1}t}v_{1}+c_{2}e^{\lambda_{2}t}v_{2}$ where **v**_1, 2_ are the eigenvectors of λ_1, 2_, *c*_1, 2_ are constants determined by initial conditions, and *t* is time. So the shape of local trajectories near a fixed point, and thus its stability type, is determined by the signs of T, Δ, and T^2^ − 4Δ. A fixed point is

- a stable node if Δ > 0, T^2^ − 4Δ > 0, and T < 0,
- a stable spiral if Δ > 0, T^2^ − 4Δ < 0, and T < 0,
- an unstable node if Δ > 0, T^2^ − 4Δ > 0, and T > 0,
- an unstable spiral if Δ > 0, T^2^ − 4Δ < 0, and T > 0, or
- a saddle point if Δ < 0 (see Strogatz, 1994).

We categorize the driven behavior of a canonical oscillator by examining how the stability of fixed point(s) varies for different values of forcing parameters Ω and *F*. Stability analysis is done for four regimes of intrinsic behavior using representative parameter settings (see Figure 1): critical Hopf (α = 0, β_1_ < 0, β_2_ = 0), supercritical Hopf (α > 0, β_1_ < 0, β_2_ = 0), supercritical double limit cycle (α < 0, β_1_ > 0, β_2_ < 0, local max > 0), and subcritical double limit cycle regimes (α < 0, β_1_ > 0, β_2_ < 0, local max < 0).

## 3. Results

### 3.1. Critical hopf oscillator

A critical Hopf oscillator is a canonical oscillator poised at the critical point of a supercritical Hopf bifurcation (where α = 0 and β_1_ < 0), which means the system is on the verge of spontaneous oscillation. Oscillatory instability at a Hopf bifurcation has recently been shown to underlie non-linear cochlear dynamics characterized by frequency selectivity, sensitivity to weak signals, and non-linear compression (see Hudspeth et al., 2010, for a review), and a bank of oscillators poised at or near Hopf bifurcation points has been used as a model of the cochlea (Jülicher et al., 2001; Duke and Jülicher, 2003; Kern and Stoop, 2003; Magnasco, 2003; Stoop et al., 2005). Here, we choose the simple parameter setting of α = 0, β_1_ < 0, and β_2_ = 0 to analyze the behavior of a critical Hopf oscillator under sinusoidal forcing, but there are other parameter regimes that share qualitatively the same driven dynamics (e.g., α, β_1_, β_2_ < 0; see Section 3.5 for a classification of parameter regimes by driven behavior).

**Figure 3:**
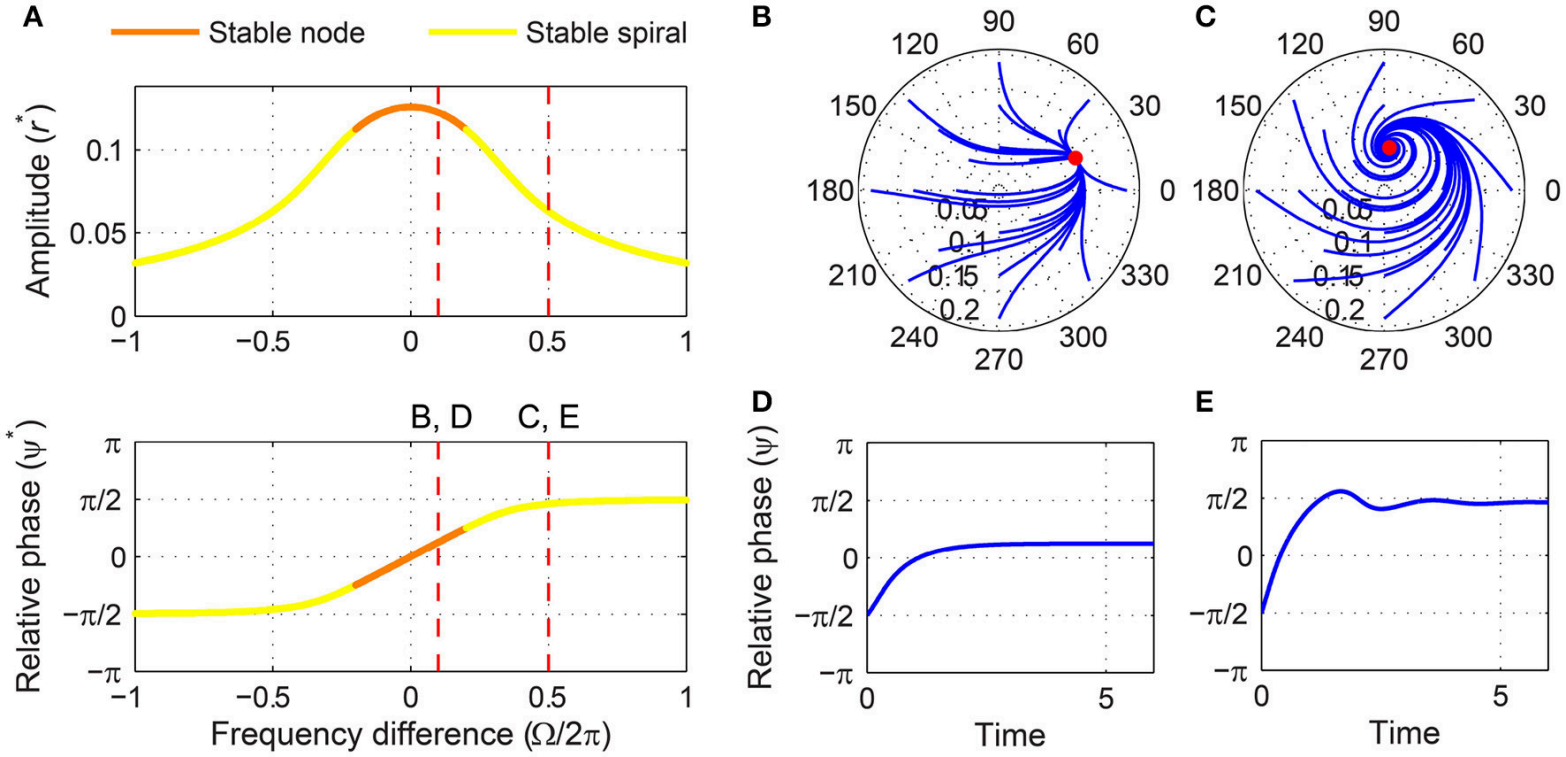
**Driven behavior of a critical Hopf oscillator**. **(A)** Steady-state amplitude and relative phase as a function of frequency difference (α = 0, β_1_ = −100, β_2_ = 0, *F* = 0.2), with vertical dashed lines indicating the frequency differences used for **(B–E)**, **(B)** trajectories attracted to a stable node in the (*r*, ψ) plane starting from a set of different initial conditions (Ω∕2π = 0.1), **(C)** trajectories attracted to a stable spiral (Ω∕2π = 0.5), **(D)** relative phase plotted over time for a trajectory in **(B)** (phase locking), and **(E)** relative phase plotted over time for a trajectory in **(C)** (phase locking). Filled circles in **(B,C)** indicate stable fixed points.

A stability analysis shows that a critical Hopf oscillator phase-locks to sinusoidal forcing of any frequency and amplitude. For a fixed forcing amplitude, the steady-state amplitude r^*^ is maximum when the forcing frequency is the same as the natural frequency (i.e., Ω = 0), for which the steady-state relative phase ψ^*^ is zero indicating in-phase synchronization (Figure 3A). As the natural frequency and the forcing frequency become more different, r^*^ decreases monotonically and approaches zero while ψ^*^ approaches $\pm \frac{\pi }{2}$. While the fixed point (r^*^, ψ^*^) remains stable for all values of Ω, it changes its stability type from a stable node to a stable spiral as |Ω| increases from 0. It is clearly seen in the (*r*, ψ) space that the two attractors have distinct local trajectories (Figures 3B,C). The way *r* and ψ approach their steady-state values in time (monotonic vs. oscillating approach) reflects the difference between a node and a spiral (only the relative phase is shown in Figures 3D,E).

We can find the boundary between stable nodes and stable spirals by solving T^2^ − 4Δ = 0, *ṙ* = 0, and $\dot{\psi }=0$ simultaneously where T and Δ are the trace and determinant of the Jacobian matrix evaluated at a fixed point (see Section 2). We find that the boundary is at

$$|\Omega_{c}|=\sqrt[{3}]{-\frac{\beta_{1}F^{2}}{2}},$$

for which

$$\begin{array}{l} \left(r_{c}^{*},\psi_{c}^{*}\right)=\left(\sqrt[{6}]{\frac{F^{2}}{2\beta_{1}^{2}}},\pm \frac{\pi }{4}\right). \end{array}$$

**Figure 4:**
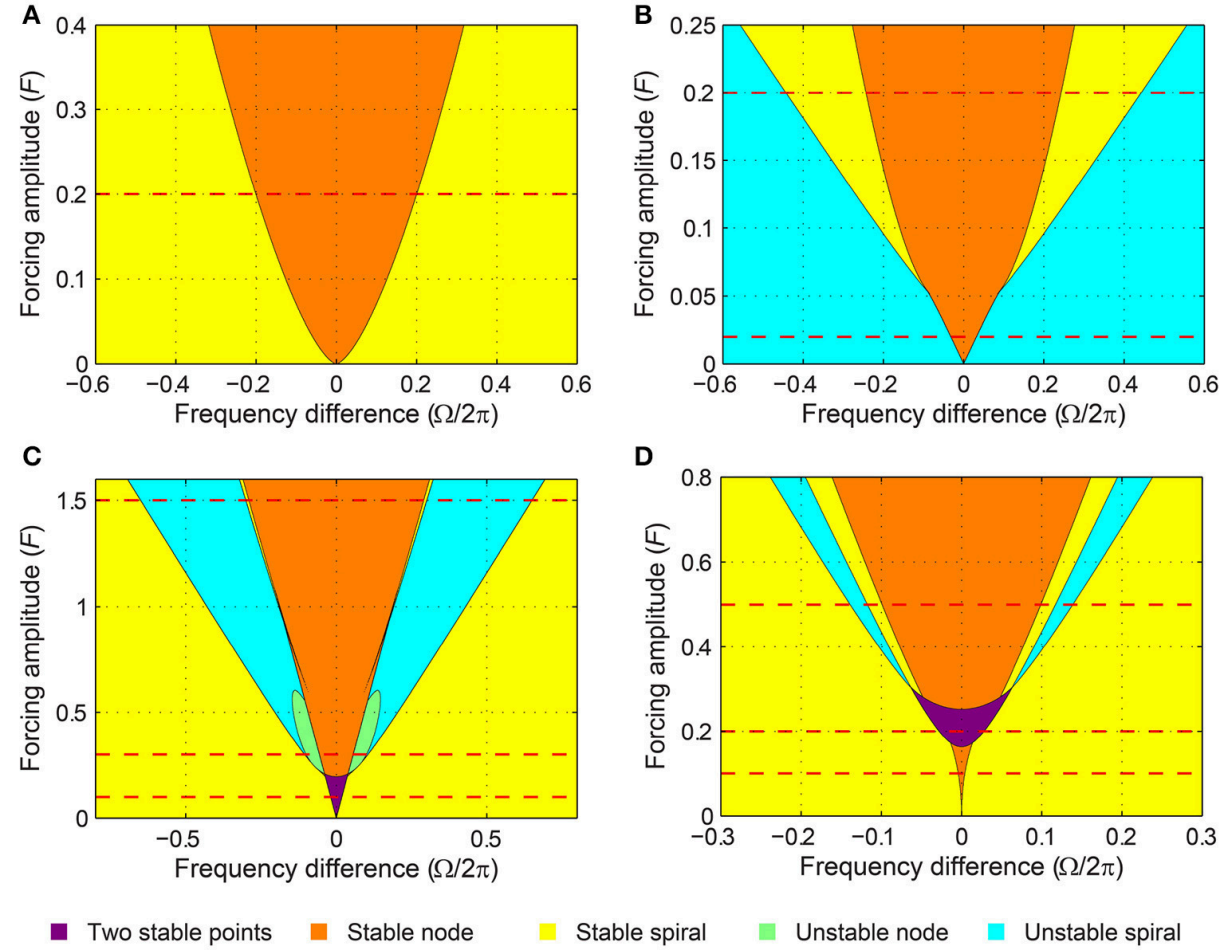
**Stability regions for a canonical oscillator under sinusoidal forcing**. The stability of driven state (r^*^, ψ^*^) is shown as a function of forcing amplitude and frequency difference for **(A)** a critical Hopf oscillator (α = 0, β_1_ = −100, β_2_ = 0), **(B)** a supercritical Hopf oscillator (α = 1, β_1_ = −100, β_2_ = 0), **(C)** a supercritical double limit cycle oscillator (α = −1, β_1_ = 4, β_2_ = −1, ϵ = 1), and **(D)** a subcritical double limit cycle oscillator (α = −1, β_1_ = 2.5, β_2_ = −1, ϵ = 1). The color indicates the stability type of a stable fixed point if there is one (purple if there are two). If there is no stable fixed point, the color indicates the stability of an unstable fixed point. Dashed horizontal lines indicate the forcing amplitudes used for Figures 3, 5–**11**. See also Supplementary Videos 1–4.

A critical Hopf oscillator shows the same set of driven behaviors summarized in Figure 3 for all levels of forcing amplitude. With increasing *F*, r^*^ increases and the node-spiral boundary widens, but no qualitatively different behaviors are introduced as the forcing amplitude changes (Figure 4A, see also Supplementary Video 1).

### 3.2. Supercritical hopf oscillator

A supercritical Hopf oscillator, which is on the supercritical branch of a supercritical Hopf bifurcation (α > 0, β_1_ < 0), has a non-zero spontaneous amplitude (Figure 1B) and has been used as a model of spontaneously oscillating systems such as haircell bundles (Fredrickson-Hemsing et al., 2012). (Spontaneous amplitude refers to the steady-state amplitude of an oscillator when no external forcing is applied). A stability analysis shows that it has two distinct sets of driven behaviors depending on the forcing amplitude and that, unlike a critical Hopf oscillator, it does not always phase-lock to sinusoidal forcing.

**Figure 5:**
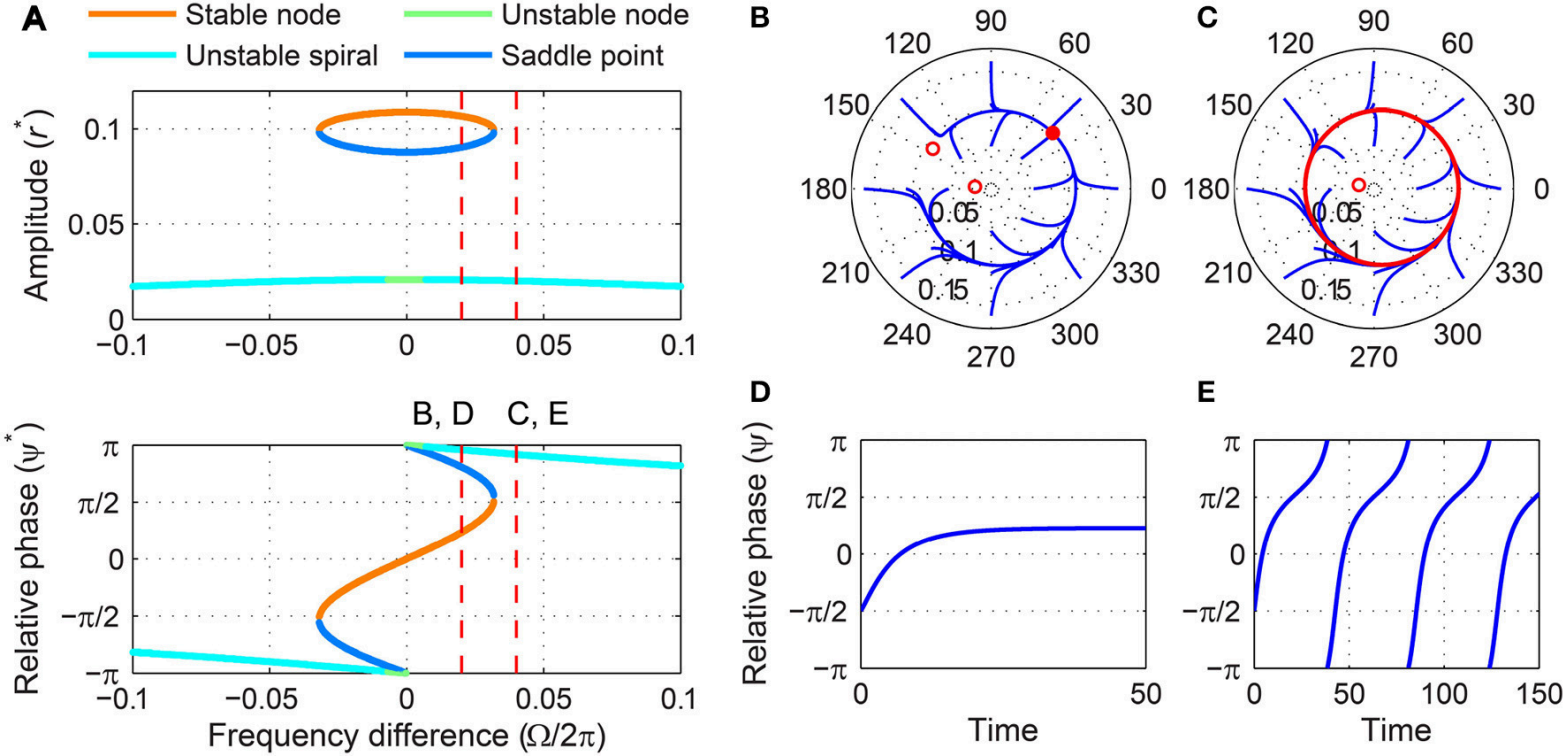
**Driven behavior of a supercritical Hopf oscillator under weak forcing**. **(A)** Steady-state amplitude and relative phase as a function of frequency difference (α = 1, β_1_ = −100, β_2_ = 0, *F* = 0.02), with vertical dashed lines indicating the frequency differences used for **(B–E)**, **(B)** trajectories attracted to a stable node in the (*r*, ψ) plane (Ω∕2π = 0.02), **(C)** trajectories drawn to a limit cycle (Ω∕2π = 0.04), **(D)** relative phase plotted over time for a trajectory in **(B)** (phase locking), and **(E)** relative phase plotted over time for a trajectory in **(C)** (phase slip). In **(B,C)**, filled and empty circles indicate stable and unstable fixed points respectively, and red lines show limit-cycle orbits.

For weak forcing, there exist three steady-state solutions for small frequency differences, two of which are a saddle-node pair, and just one unstable solution for large frequency differences (Figure 5A). As the frequency difference increases from zero for a fixed forcing amplitude, the saddle and node are lost via a saddle-node invariant-circle (SNIC) bifurcation (also called a saddle-node infinite-period or SNIPER bifurcation), which leaves a stable (attracting) limit-cycle orbit with an unstable fixed point inside (Figures 5A–C). The critical frequency difference for which a SNIC bifurcation occurs can be obtained by solving Δ = 0, *ṙ* = 0, and $\dot{\psi }=0$ together. We find the SNIC boundary to be at

$$\begin{array}{l} \Gamma_{SN}=|\Omega_{c}|=\sqrt{-\left(\alpha +3\beta_{1}r_{c}^{*2}\right)\left(\alpha +\beta_{1}r_{c}^{*2}\right)}, \end{array}$$

where $r_{c}^{*}$ is the bigger of the two positive real roots of $2\beta_{1}^{2}r_{c}^{*6}+2\alpha \beta_{1}r_{c}^{*4}+F^{2}=0$. For |Ω| < Γ_*SN*_, the canonical oscillator phase-locks to the forcing, with its driven state attracted to a stable node (Figures 5B,D). For |Ω| > Γ_*SN*_ for which only one unstable fixed point exists, the relative phase does not converge to a steady-state value but makes full 2π-rotations (i.e., phase slip), meaning the oscillator is not phase-locked to the input, and the amplitude fluctuates near the spontaneous amplitude (Figures 5C,E). (The spontaneous amplitude of the oscillator shown in Figure 5 is $\sqrt{-\alpha ∕\beta_{1}}=0.1$). Thus, the SNIC bifurcation marks the phase-locking boundary for a supercritical Hopf oscillator under weak forcing. Note that the flow of relative phase is slow near $\frac{\pi }{2}$ (or $-\frac{\pi }{2}$ when Ω < 0) where the saddle-node pair collides and leaves a bottleneck or a “ghost” (Figure 5E).

**Figure 6:**
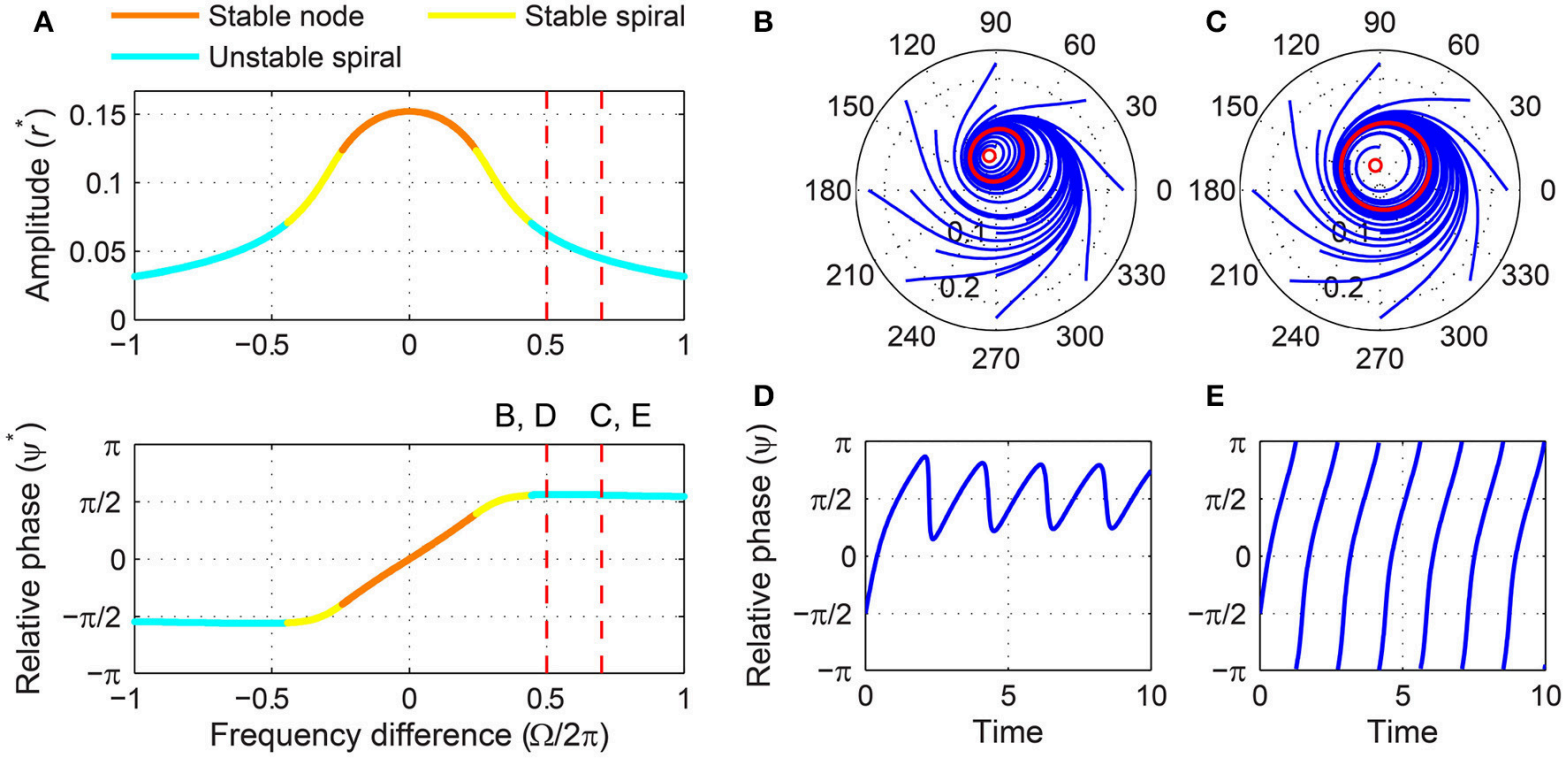
**Driven behavior of a supercritical Hopf oscillator under strong forcing**. **(A)** Steady-state amplitude and relative phase as a function of frequency difference (α = 1, β_1_ = −100, β_2_ = 0, *F* = 0.2), with vertical dashed lines indicating the frequency differences used for **(B–E)**, **(B)** trajectories attracted to a phase-trapped libration in the (*r*, ψ) plane (Ω∕2π = 0.5), **(C)** trajectories attracted to a rotation (Ω∕2π = 0.7), **(D)** relative phase plotted over time for a trajectory in **(B)** (phase-trapped frequency locking without phase locking), and **(E)** relative phase plotted over time for a trajectory in **(C)** (phase slip). In **(B,C)**, empty circles indicate unstable fixed points, and red lines show limit-cycle orbits.

For stronger forcing, only one fixed point exists for all values of frequency difference, but it changes from a stable node to a stable spiral then to an unstable spiral as |Ω| grows from zero (Figure 6A). Now the phase-locking boundary is at the transition from a stable spiral to an unstable spiral (i.e., a Hopf bifurcation in the (*r*, ψ) space), which we can find by solving T = 0, *ṙ* = 0, and $\dot{\psi }=0$ together. We get

$$\begin{array}{l} \Gamma_{H}=|\Omega_{c}|=\sqrt{-\frac{2\beta_{1}F^{2}}{\alpha }-\frac{\alpha^{2}}{4}}, \end{array}$$

for which

$$\begin{array}{l} r_{c}^{*}=\sqrt{-\frac{\alpha }{2\beta_{1}}}\;\text{and}\;\cos\psi_{c}^{*}=-\frac{1}{F}\sqrt{-\frac{\alpha^{3}}{8\beta_{1}}}. \end{array}$$

Note that at the Hopf boundary the steady-state amplitude $r_{c}^{*}$ is smaller than the spontaneous amplitude ($r^{*}=\sqrt{-\alpha ∕\beta_{1}}$ when *F* = 0), and the steady-state relative phase $\psi_{c}^{*}$ goes beyond $\pm \frac{\pi }{2}$ since $\cos\psi_{c}^{*}$ is negative (see also Figure 6A).

Outside the phase-locking range for strong forcing, the driven behavior of a supercritical Hopf oscillator can be divided into two categories. Just outside the Hopf boundary, the driven state (*r*, ψ) circles on a stable limit cycle which forms around the unstable spiral and is small enough not to encompass the origin (Figure 6B). In this case, the relative phase changes over time but is bounded and does not traverse the full 2π range (Figure 6D), which is called a libration (as opposed to a rotation, see Strogatz, 1994). When averaged over time, this “phase-trapped” oscillation has the same mean frequency as the input frequency, so it can be described as frequency locking without phase locking (Hoppensteadt and Izhikevich, 1997; Pikovsky et al., 2000, 2001). As |Ω| increases further, the limit cycle around the unstable spiral grows and eventually encompasses the origin (Figure 6C), and the relative phase starts making full rotations (Figure 6E). At this point, the average instantaneous frequency of the oscillator is different from the input frequency and approaches the natural frequency as |Ω| approaches infinity.

The existence of phase-trapped libration (thus, frequency locking) outside the phase-locking boundary is a distinct feature of the Hopf boundary. When crossing the SNIC boundary for weak forcing, the driven state changes directly from phase locking to phase slipping (Figures 5B–E). The same transition of driven behaviors is found for phase models (i.e., oscillators described by their phases only). This is not unexpected since under weak forcing the amplitude of a supercritical Hopf oscillator is effectively constant and does not change much from its spontaneous value.

The SNIC phase-locking boundary and the Hopf boundary exist only for weak and strong forcing levels respectively (Figure 4B; see also Supplementary Video 2 for the transition between Figures 5A, 6A), but the two types of phase-locking boundary coexist for a small range of intermediate forcing level. The SNIC boundary exists for forcing amplitudes smaller than

$$\begin{array}{l} F_{SN}=\sqrt{-\frac{8\alpha^{3}}{27\beta_{1}}}, \end{array}$$

for which the fixed point at the SNIC bifurcation is

$$\begin{array}{l} \left(r_{c}^{*},\psi_{c}^{*}\right)=\left(\sqrt{-\frac{2\alpha }{3\beta_{1}}},\pm \frac{2\pi }{3}\right). \end{array}$$

The Hopf boundary, on the other hand, exists for forcing amplitudes greater than

$$\begin{array}{l} F_{H}=\sqrt{-\frac{\alpha^{3}}{4\beta_{1}}}, \end{array}$$

for which the fixed point at the bifurcation is

$$\begin{array}{l} \left(r_{c}^{*},\psi_{c}^{*}\right)=\left(\sqrt{-\frac{\alpha }{2\beta_{1}}},\pm \frac{3\pi }{4}\right). \end{array}$$

Note that *F*_*SN*_ > *F*_*H*_. For forcing amplitudes between the two values, two stable fixed points (a stable node and a stable spiral) coexist for some values of Ω near the locking boundaries. Also, this region of the forcing parameter space (i.e., near the tip of the yellow regions in Figure 4B) contains a complicated but well-studied set of bifurcations. For instance, a Bogdanov–Takens bifurcation is at the lower end of Γ_*H*_, and a cusp point is at the upper end of Γ_*SN*_. Detailed analysis of the dynamics around them can be found in Guckenheimer and Holmes (1983), for example. Also, it is worth noting that the same set of bifurcations are found for other periodically driven non-linear oscillators or populations of oscillators such as the forced van der Pol oscillator (Holmes and Rand, 1978) and the forced Kuramoto model (Childs and Strogatz, 2008). However, the canonical model analyzed here, with its simple mathematical form, allows closer analytical examination than is possible for more complex models.

### 3.3. Supercritical double limit cycle oscillator

As shown in the introduction, a supercritical DLC oscillator (α < 0, β_1_ > 0, β_2_ < 0, local max > 0) has two stable autonomous behaviors. Depending on the initial condition, it can be attracted to an equilibrium at zero or oscillate spontaneously with non-zero amplitudes at its natural frequency (Figure 1C). The transition between a supercritical DLC oscillator and a subcritical DLC oscillator, called a double limit cycle bifurcation or a fold limit cycle bifurcation, has been suggested to be involved in several types of bursting neurons (Izhikevich, 2000, 2001). When driven by a sinusoid, a supercritical DLC oscillator shows three distinct sets of behaviors depending on the strength of the forcing, and many of these behaviors involve bistability as well.

**Figure 7:**
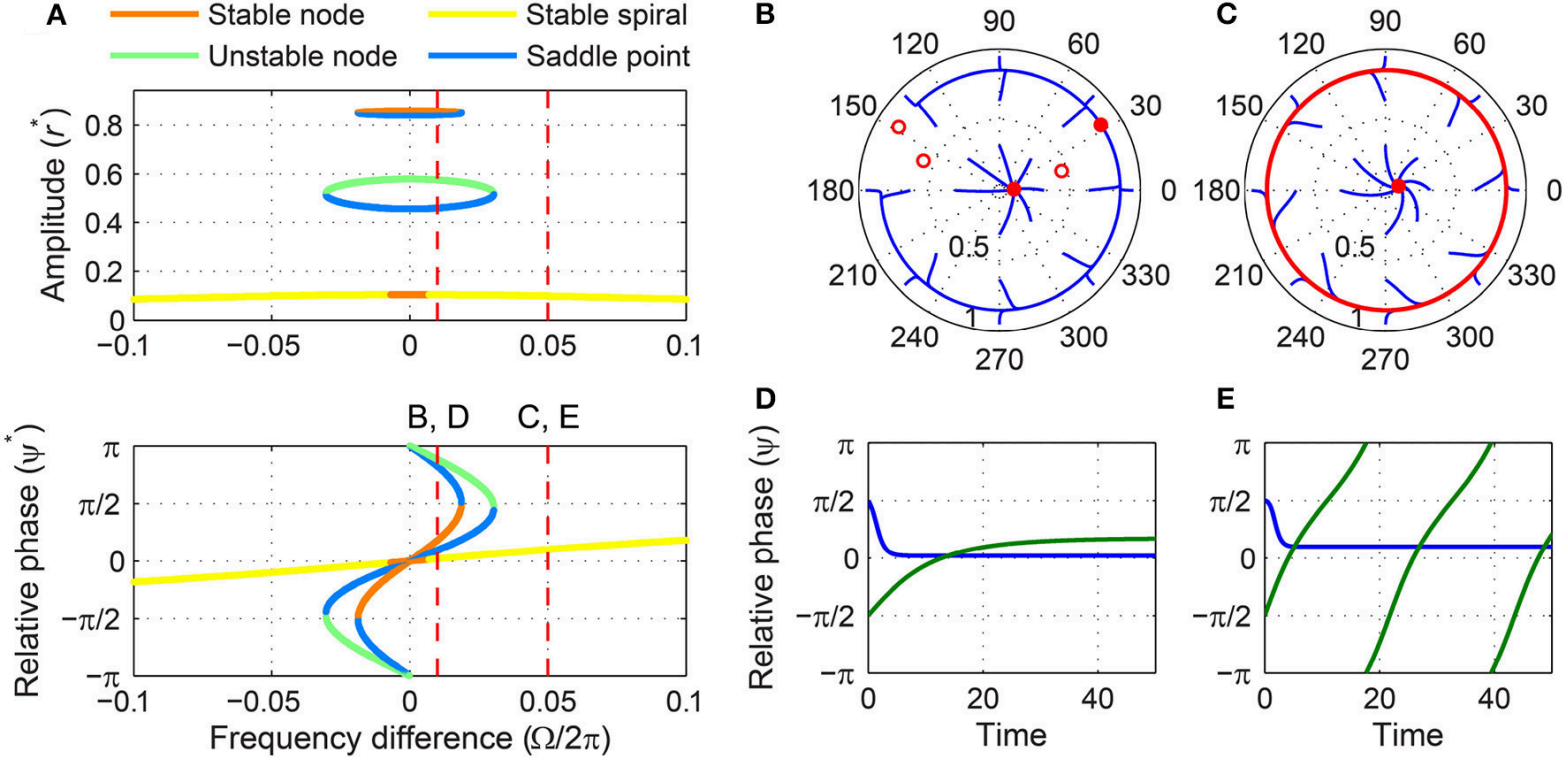
**Driven behavior of a supercritical double limit cycle oscillator under weak forcing**. **(A)** Steady-state amplitude and relative phase as a function of frequency difference (α = −1, β_1_ = 4, β_2_ = −1, ϵ = 1, *F* = 0.1), with vertical dashed lines indicating the frequency differences used for **(B–E)**, **(B)** trajectories attracted to either of two stable fixed points in the (*r*, ψ) plane (Ω∕2π = 0.01), **(C)** trajectories drawn to either a stable spiral or a limit-cycle rotation (Ω∕2π = 0.05), **(D)** relative phase plotted over time for two trajectories in **(B)** (both phase locking), and **(E)** relative phase plotted over time for two trajectories in **(C)** (phase locking in blue, phase slip in green). In **(B,C)**, filled and empty circles indicate stable and unstable fixed points respectively, and red lines show limit-cycle orbits.

Under weak forcing, it has two stable fixed points for small frequency differences and only one for large frequency differences (Figure 7A). The stable fixed point with a small amplitude exists for all values of Ω, but the one with a high amplitude (a stable node) is lost via a SNIC bifurcation and leaves a limit-cycle rotation (phase slip) where it collides with a saddle point (Figures 7B–E). (Due to non-zero β_2_ and the higher-order terms it introduces, it is not possible to get a closed-form expression for the frequency difference for which a bifurcation of driven states occurs, as was done for the models with β_2_ = 0 discussed above. However, the location of bifurcations can be obtained using numerical methods.) The transition from phase locking on a stable node to phase slip on a stable limit cycle is identical to what happens at the phase-locking boundary of a supercritical Hopf oscillator under weak forcing (Figure 5), but the presence of a second stable fixed point at low amplitudes differentiates supercritical DLC oscillators from supercritical Hopf oscillators. So, a weakly forced supercritical DLC oscillator shows bistability for all values of frequency difference—phase locking at high or low amplitudes for small values of |Ω|, and phase locking at low amplitudes or phase slip at high amplitudes for large values of |Ω|—and the initial condition determines the driven state to which the oscillator is attracted.

**Figure 8:**
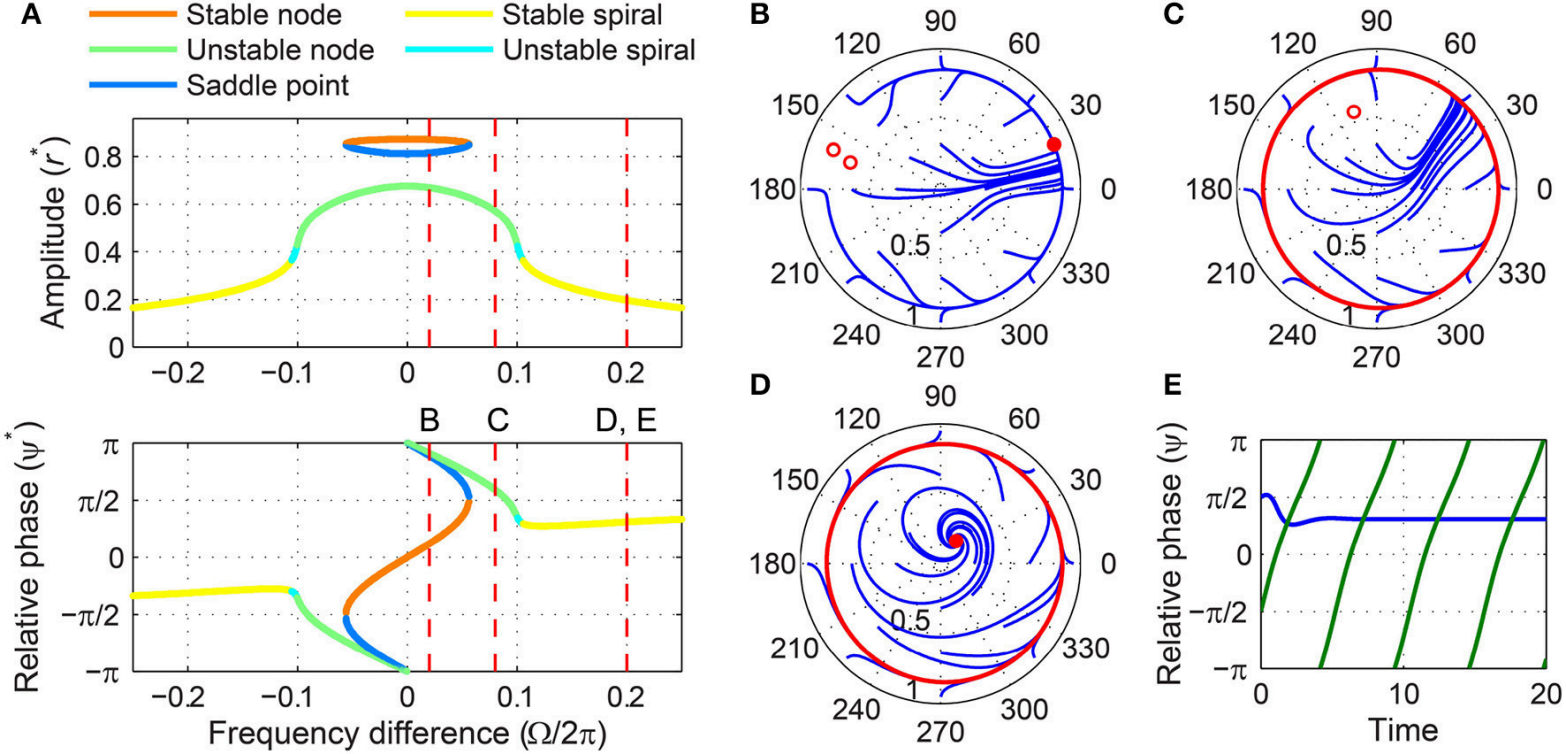
**Driven behavior of a supercritical double limit cycle oscillator under intermediate forcing**. **(A)** Steady-state amplitude and relative phase as a function of frequency difference (α = −1, β_1_ = 4, β_2_ = −1, ϵ = 1, *F* = 0.3), with vertical dashed lines indicating the frequency differences used for **(B–E)**, **(B)** trajectories attracted to a stable node in the (*r*, ψ) plane (Ω∕2π = 0.02), **(C)** trajectories drawn to a limit-cycle rotation (Ω∕2π = 0.08), **(D)** trajectories drawn to either a stable spiral or a limit cycle (Ω∕2π = 0.2), and **(E)** relative phase plotted over time for two trajectories in **(D)** (phase locking in blue, phase slip in green). In **(B–D)**, filled and empty circles indicate stable and unstable fixed points respectively, and red lines show limit-cycle orbits.

For intermediate forcing amplitudes, the saddle-node pair at high amplitudes still exists for small frequency differences, but the stable fixed point at low amplitudes exists only for large frequency differences (Figure 8A). There is a range of intermediate frequency differences for which no stable fixed point exists and all trajectories are attracted to a limit-cycle rotation that the saddle-node pair leaves (Figure 8C). As the frequency difference increases, the fixed point inside the limit cycle changes from an unstable node to an unstable spiral and eventually to a stable spiral (Figures 8A,C,D). The emergence of a stable spiral inside a stable (attracting) limit cycle indicates a subcritical Hopf bifurcation. Thus, as the frequency difference increases from zero, the driven state of a supercritical DLC oscillator under intermediate forcing goes from phase locking (a stable node, Figure 8B) to phase slip (a stable limit cycle, Figure 8C) and then to the bistability between phase locking and phase slip (a stable spiral inside a stable limit cycle, Figure 8D).

**Figure 9:**
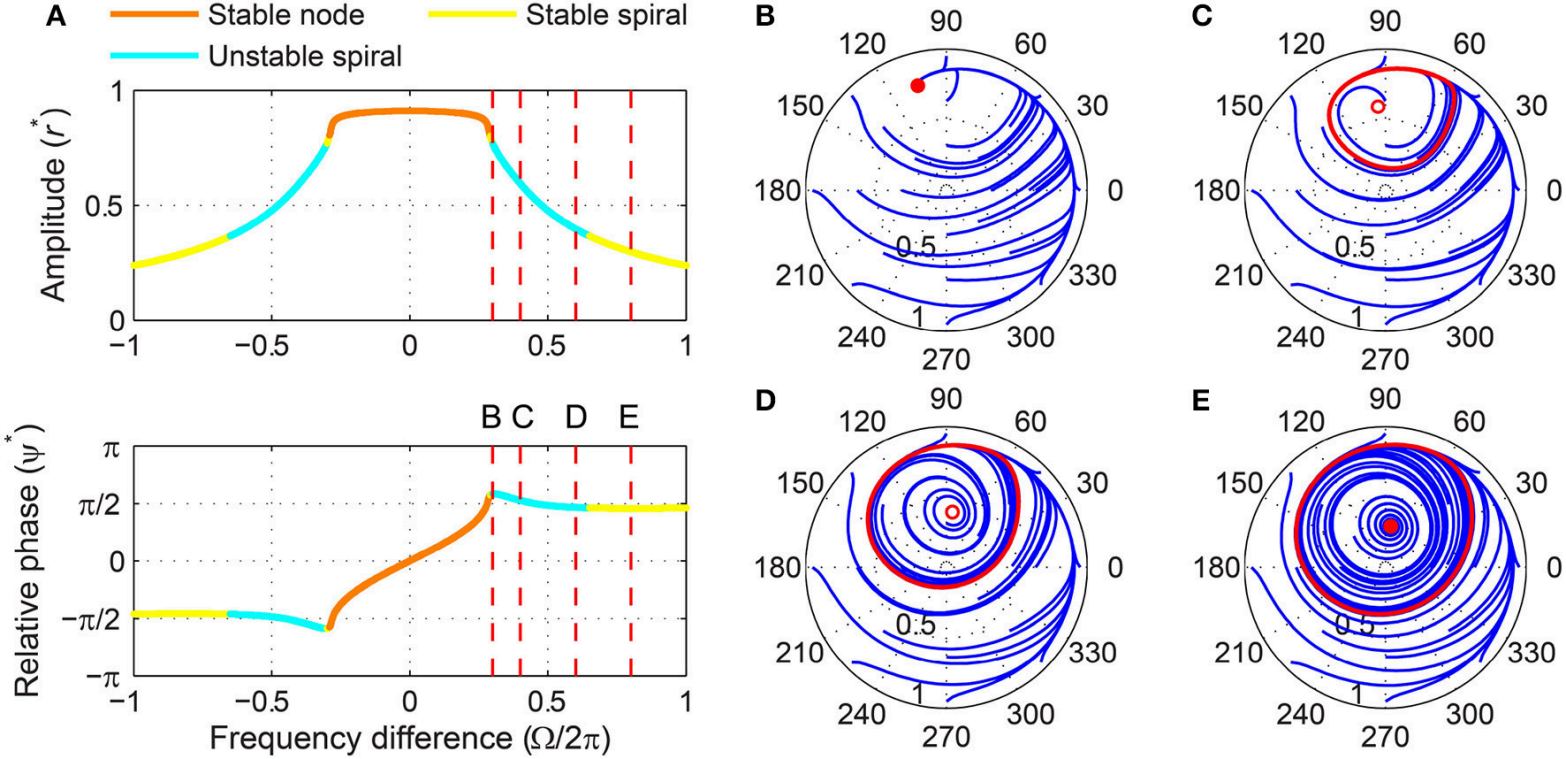
**Driven behavior of a supercritical double limit cycle oscillator under strong forcing**. **(A)** Steady-state amplitude and relative phase as a function of frequency difference (α = −1, β_1_ = 4, β_2_ = −1, ϵ = 1, *F* = 1.5), with vertical dashed lines indicating the frequency differences used for **(B–E)**, **(B)** trajectories attracted to a stable spiral in the (*r*, ψ) plane (Ω∕2π = 0.3, phase locking), **(C)** trajectories drawn to a phase-trapped libration (Ω∕2π = 0.4, frequency locking), **(D)** trajectories drawn to a rotation (Ω∕2π = 0.6, phase slip), and **(E)** trajectories drawn to either a stable spiral or a rotation (Ω∕2π = 0.8, phase locking and phase slip). In **(B–E)**, filled and empty circles indicate stable and unstable fixed points respectively, and red lines show limit-cycle orbits.

When the forcing amplitude is further increased, only one fixed point exists for any value of frequency difference (Figure 9A). Similar to a strongly forced supercritical Hopf oscillator (Figure 6), the driven state (*r*, ψ) of a strongly forced supercritical DLC oscillator goes through transitions from a stable node (phase locking), a stable spiral (phase locking, Figure 9B), a libration around an unstable spiral (frequency locking without phase locking, Figure 9C), and a rotation around an unstable spiral (phase slip, Figure 9D). In addition to these states, a supercritical DLC oscillator exhibits another driven behavior for even larger frequency differences, bistability between phase locking on a stable spiral and phase slip on a stable limit cycle (Figure 9E). So, a strongly forced supercritical DLC oscillator has two phase-locking boundaries, a supercritical Hopf bifurcation (between Figure 9B and Figure 9C) and a subcritical Hopf bifurcation (between Figure 9D and Figure 9E). Figure 4C and Supplementary Video 3 show how the three sets of driven behaviors shown in Figures 7–9 transition between each other.

### 3.4. Subcritical double limit cycle oscillator

Like a critical Hopf oscillator, a subcritical DLC oscillator is attracted to an equilibrium at zero when it is not driven (Figure 1D). But the presence of a local maximum in the amplitude vector field makes its driven dynamics more varied and interesting than that of a critical Hopf oscillator. Like a supercritical DLC oscillator, a subcritical DLC oscillator exhibits three different sets of driven behaviors depending on the forcing amplitude.

**Figure 10:**
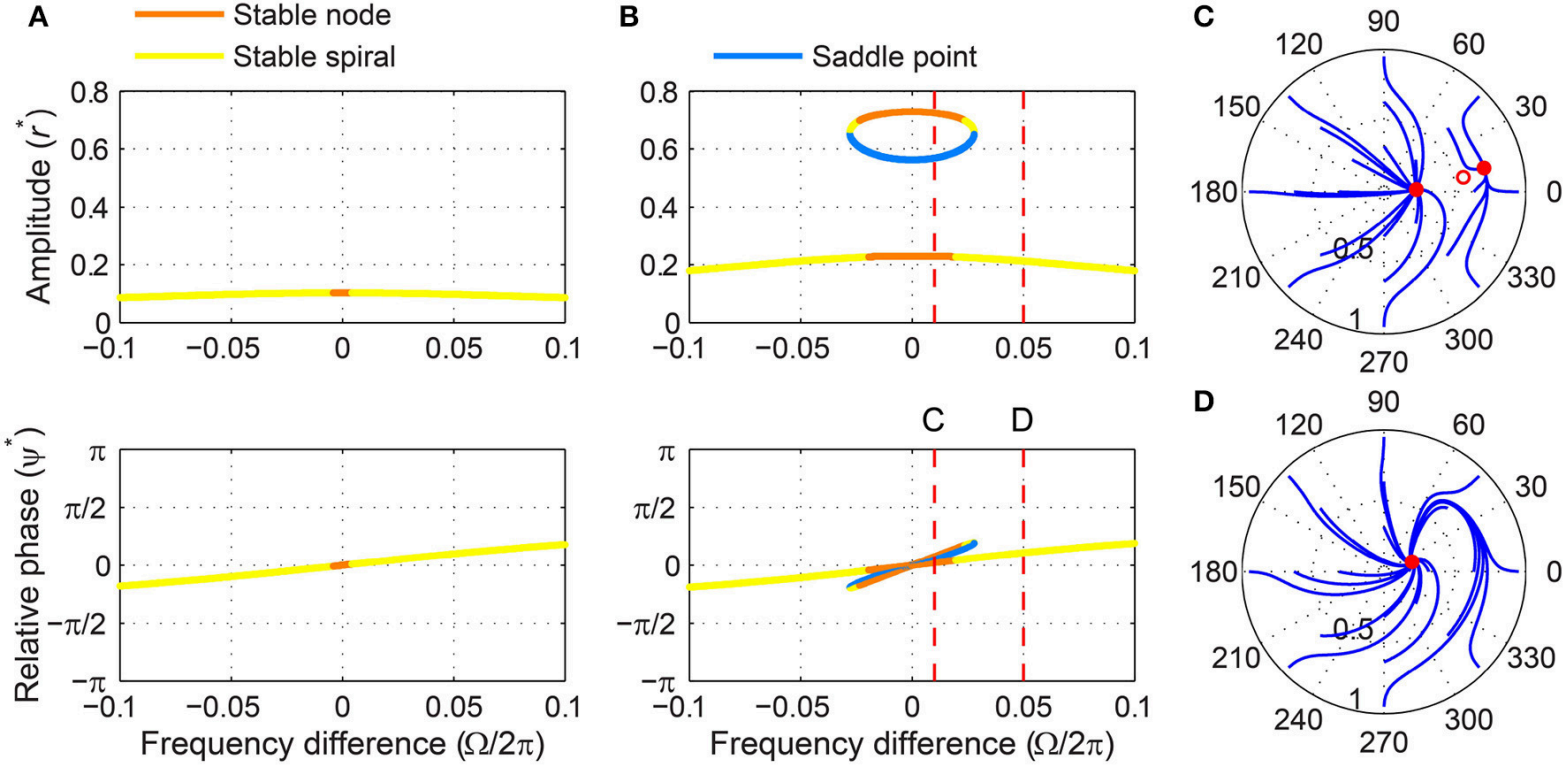
**Driven behavior of a subcritical double limit cycle oscillator under weak and intermediate forcing**. **(A)** Steady-state amplitude and relative phase as a function of frequency difference for weak forcing (α = −1, β_1_ = 2.5, β_2_ = −1, ϵ = 1, *F* = 0.1) and **(B)** for intermediate forcing (*F* = 0.2), with vertical dashed lines indicating the frequency differences used for **(C,D)**, **(C)** trajectories attracted to either of two stable fixed points in the (*r*, ψ) plane (Ω∕2π = 0.01, both phase locking), and **(D)** trajectories drawn to a stable spiral (Ω∕2π = 0.05, phase locking). In **(C,D)**, filled and empty circles indicate stable and unstable fixed points respectively.

For weak forcing, it behaves like a critical Hopf oscillator, with its driven state attracted to a stable node when |Ω| is small and to a stable spiral when |Ω| is large (Figure 10A). For intermediate forcing amplitudes, a pair of fixed points appears at high amplitudes and they are lost via a saddle-node bifurcation at a certain frequency difference (Figures 10B–D). (It is not clearly seen in Figure 10B, but the stable fixed point turns back into a stable node just before it collides with the saddle point.) Note that this saddle-node bifurcation does not leave a limit cycle like a SNIC bifurcation (Figure 10D; compare with Figure 7C), making the fixed point at low amplitudes the only (global) attractor. This means that bistability exists only for small frequency differences for a subcritical DLC oscillator under intermediate forcing.

**Figure 11:**
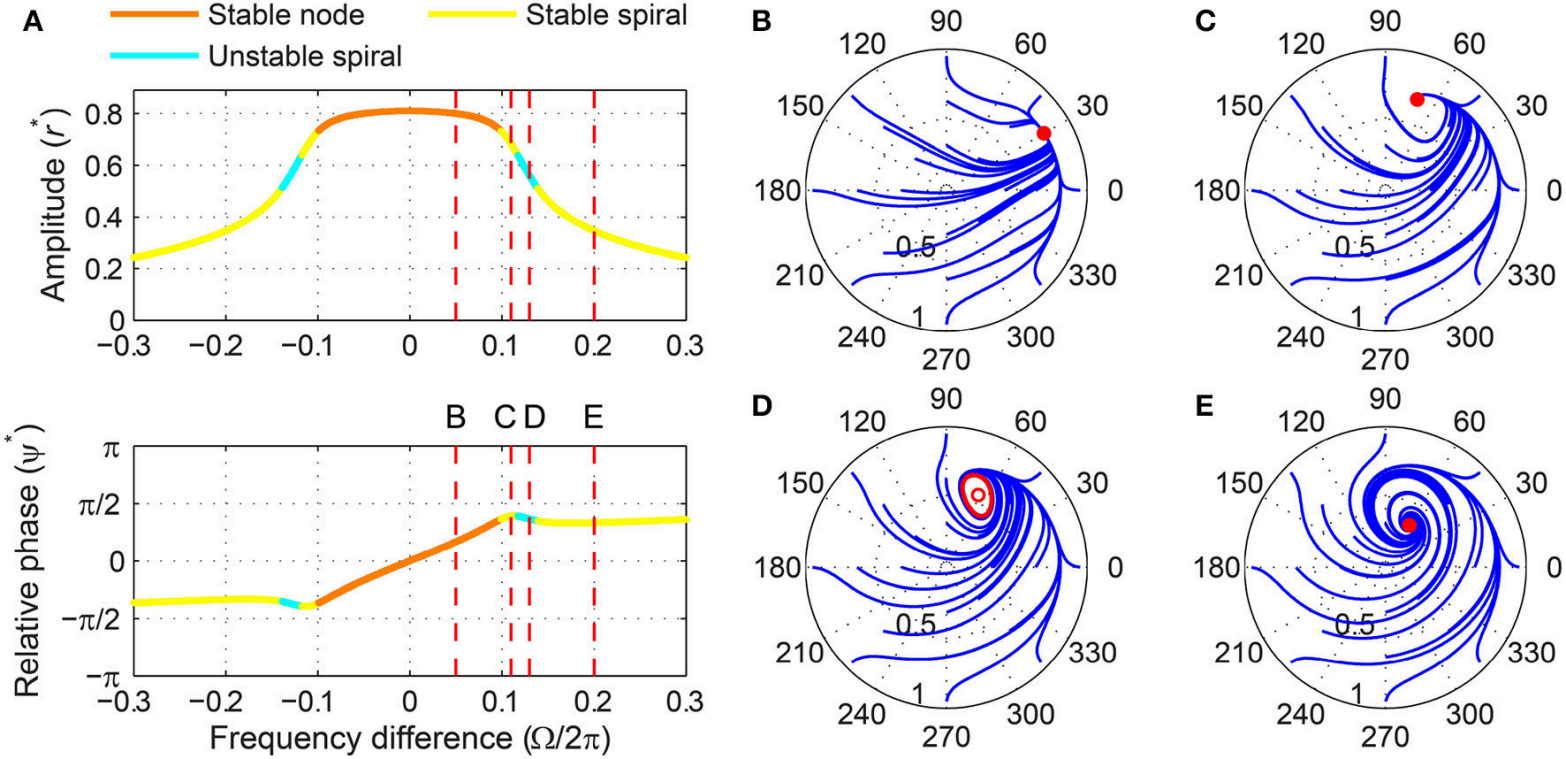
**Driven behavior of a subcritical double limit cycle oscillator under strong forcing**. **(A)** Steady-state amplitude and relative phase as a function of frequency difference (α = −1, β_1_ = 2.5, β_2_ = −1, ϵ = 1, *F* = 0.5), with vertical dashed lines indicating the frequency differences used for **(B–E)**, **(B)** trajectories attracted to a stable node in the (*r*, ψ) plane (Ω∕2π = 0.05, phase locking), **(C)** trajectories attracted to a stable spiral (Ω∕2π = 0.11, phase locking), **(D)** trajectories drawn to a phase-trapped libration (Ω∕2π = 0.13, frequency locking), and **(E)** trajectories drawn to a stable spiral (Ω∕2π = 0.2, phase locking). In **(B–E)**, filled and empty circles indicate stable and unstable fixed points respectively, and red lines show limit-cycle orbits.

When driven strongly, a subcritical DLC oscillator has the same set of fixed points as a supercritical DLC oscillator—a stable node, a stable spiral, an unstable spiral, and a stable spiral as |Ω| increases from zero (compare Figure 9A and Figure 11A). A supercritical Hopf bifurcation occurs at the first phase-locking boundary, where a stable spiral turns unstable and a stable limit cycle grows around it (between Figure 11C and Figure 11D). However, the limit cycle does not grow into a rotation that encompasses the origin, which is the case for a supercritical DLC oscillator. Instead, it shrinks back and turns into a stable spiral via another supercritical Hopf bifurcation (between Figure 11D and Figure 11E). In the absence of a SNIC or subcritical Hopf bifurcation, a strongly driven subcritical DLC oscillator shows no bistability and, since the only non-locked behavior is a libration (Figure 11D), it either phase-locks or frequency-locks to the input for all values of Ω. The transitions between different forcing levels are shown in Figure 4D and Supplementary Video 4.

### 3.5. Classification of parameter regimes by driven behavior

Above we examined the dynamics of periodically forced canonical oscillators in four different intrinsic parameter regimes. We used a representative parameter setting for each of the four distinct driven behaviors, but there are other combinations of intrinsic parameters that show the same sets of behaviors. For instance, the same set of driven behaviors is found for α > 0, β_1_ < 0, and β_2_ = 0 (discussed as a supercritical Hopf oscillator) and for α = 0, β_1_ > 0, and β_2_ < 0. This is because the two parameter settings have topologically identical autonomous amplitude vector fields (i.e., increasing from zero and then monotonically decreasing as in Figure 1B).

We can classify all possible parameter settings for canonical oscillators into four regimes with distinct driven behaviors (Table 1). Oscillators with an autonomous amplitude vector field that monotonically decreases from zero with no local extremum (Figure 1A) have the same set of driven behaviors as a critical Hopf oscillator (α = 0, β_1_ < 0, β_2_ = 0). Linear oscillators (α < 0, β_1_ = 0, β_2_ = 0) belong to this category. Oscillators whose amplitude vector fields have one local maximum (Figure 1B) have the same set of driven behaviors and bifurcations as a supercritical Hopf oscillator (α > 0, β_1_ < 0, β_2_ = 0). Oscillators with α < 0, β_1_ > 0, and β_2_ < 0 are divided into three groups depending on whether the local maximum of the amplitude vector field is above zero (Figure 1C, a supercritical DLC oscillator), is below zero (Figure 1D, a subcritical DLC oscillator), or does not exist (with the same driven behaviors as a critical Hopf oscillator).

**Table 1:** **Classification of parameter regimes by driven behavior**.

**α**	**β_1_**	**β_2_**	**Local extrema^a^**	**Discussed as**	**Bifurcations^b^**
−	0	0	None		None
0	−	0		Critical Hopf	
0	0	−			
−	−	0			
−	0	−			
0	−	−			
−	−	−			
−	+	−	(No max)		
+	−	0	One	Supercritical Hopf	SNIC (low *F*);
+	0	−			Super-Hopf (high *F*)
0	+	−			
+	−	−			
+	+	−			
−	+	−	Two (max > 0)	Supercritical DLC	SNIC (low *F*);
					SNIC, Sub-Hopf (mid *F*);
					Super-Hopf, Sub-Hopf (high *F*)
−	+	−	Two (max < 0)	Subcritical DLC	None (low *F*);
					SN (mid *F*);
					Super-Hopf, Super-Hopf (high *F*)

*SNIC, saddle-node bifurcation on an invariant circle; Super-Hopf, supercritical Hopf bifurcation; Sub-Hopf, subcritical Hopf bifurcation; SN, saddle-node bifurcation*.

a *The number of local extrema in the autonomous amplitude vector field*.

b *Bifurcations at phase-locking boundaries*.

### 3.6. Frequency scaling of logarithmic frequency networks

The analysis so far shows that for a fixed forcing amplitude the driven behavior of a canonical oscillator depends on the frequency difference Ω, not on the natural frequency ω *per se*, and is symmetrical about Ω = 0 on a linear scale (see Figure 11A, for example). This is to be expected from the fact that Equation (2), which was used for the above analysis, is not scaled by natural frequency (or time constant) as is Equation (1). Thus, the width of phase-locking range for an oscillator described by Equation (2) is constant regardless of its natural frequency if other intrinsic parameters remain the same. For an oscillator network with logarithmically equally spaced natural frequencies, this might not be desirable if we want each oscillator in the network to cover the same portion of logarithmic frequency space. Frequency scaling solves this problem by making the driven behavior depend on both frequency difference and natural frequency.

**Figure 12:**
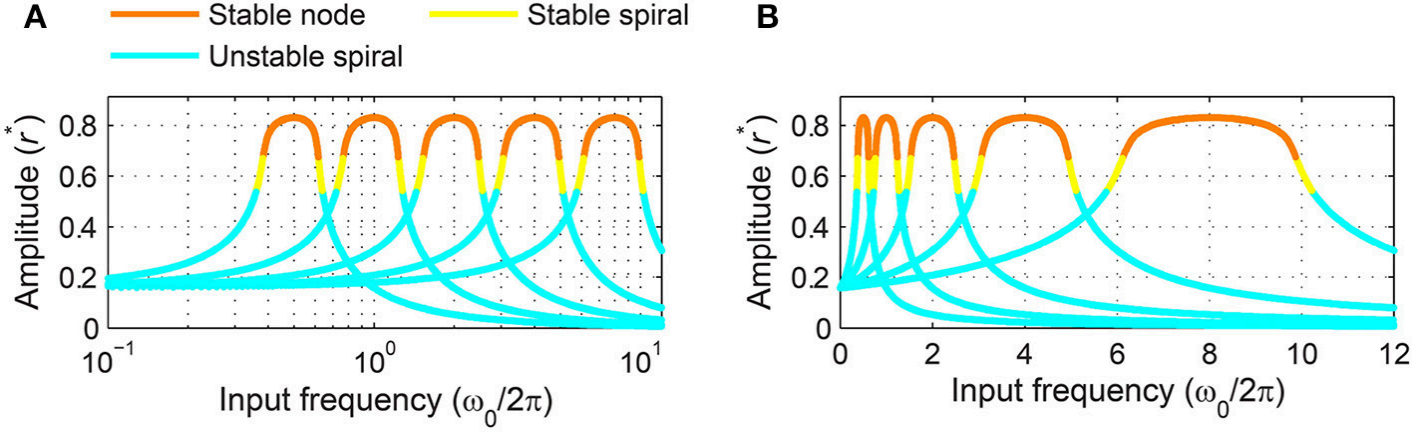
**Frequency scaled canonical oscillators in a logarithmic frequency network**. The steady-state amplitude of frequency-scaled canonical oscillators (α = 1, β_1_ = −1, β_2_ = −1, ϵ = 1, *F* = 1) with logarithmically equally spaced natural frequencies ($\frac{\omega }{2\pi }=0.5,1,2,4,8$) is plotted as a function of input frequency on **(A)** a logarithmic frequency axis and **(B)** a linear frequency axis.

The frequency-scaled version of Equation (2),

$$\begin{array}{l} \frac{1}{f}ż=z\left(\alpha +\text{i}2\pi +\beta_{1}|z{|}^{2}+\frac{\epsilon \beta_{2}|z{|}^{4}}{1-\epsilon |z{|}^{2}}\right)+Fe^{\text{i}\omega_{0}t}, \end{array}$$

has the polar form

(4) $$\left\{ \begin{array}{l} \frac{1}{f}\dot{r}=\alpha r+\beta_{1}r^{3}+\frac{\epsilon \beta_{2}r^{5}}{1-\epsilon r^{2}}+F\cos\psi \\ \frac{1}{f}\dot{\psi }=\frac{\Omega }{f}-\frac{F}{r}\sin\psi , \end{array}\right.$$

where $f=\frac{\omega }{2\pi }$ is the linear natural frequency. There are two main differences between Equation (4) and its non-scaled version, Equation (3). First, the left-hand sides of the scaled equations are multiplied by the inverse of natural frequency, indicating that high-frequency oscillators have faster dynamics and shorter relaxation time than low-frequency oscillators. Second, the right-hand sides are identical for the two versions except that the scaled version has Ω∕*f* in place of Ω in the non-scaled version. This means that the two versions share the same set of steady-state solutions and stability types, but for the frequency-scaled version the distribution of solutions in frequency space is proportional to the natural frequency. Figure 12A shows that frequency-scaled canonical oscillators of different natural frequencies have steady-state amplitude curves of an identical shape and width when plotted on a logarithmic frequency axis. But note that the amplitude curves and locking ranges are asymmetrical on the logarithmic axis because even with frequency scaling they are symmetrical in linear frequency (Figure 12B).

## 4. Discussion

Here we have examined the response of gradient frequency neural networks to periodic forcing by analyzing the driven behavior of a canonical model for such networks. Using a dynamical systems analysis of a canonical oscillator under sinusoidal forcing, we showed that oscillators with distinct autonomous behaviors have different sets of driven behaviors and different types of bifurcation at phase-locking boundaries (summarized in Table 1 and Figure 4). Oscillators that decay to zero without external forcing are found to phase-lock to sinusoidal forcing of any frequency and amplitude, if the autonomous amplitude vector field decreases monotonically (e.g., a critical Hopf oscillator). When the vector field has a below-zero local maximum (i.e., a subcritical DLC oscillator), the oscillator always phase-locks to weak forcing, shows bistability for intermediate forcing, and either phase-locks or frequency-locks to strong forcing. Oscillators with one stable non-zero spontaneous amplitude (e.g., supercritical Hopf oscillators) phase-lock only to forcing frequencies close to their natural frequencies. Just outside the phase-locking range, oscillators of this type frequency-lock to strong forcing. Finally, oscillators with two stable spontaneous amplitudes (i.e., supercritical DLC oscillators) exhibit the most diverse set of behaviors including phase locking, frequency locking, phase slip, bistability between two phase-locked states, and bistability between phase locking and phase slip. We also showed that frequency scaling makes the response of canonical oscillators constant over logarithmic frequency.

The present analysis shows how a gradient frequency network of non-linear oscillators processes a periodic signal. Given the values of intrinsic parameters and the range of natural frequencies, the analysis shows which part of the network would phase-lock to the signal while the other part oscillates near the spontaneous (i.e., autonomous) states. Thus, the response of the network as a whole would include both the signal frequency and the natural frequencies of oscillators not locked to the signal, plus some non-linear combination frequencies in non-locked, fluctuating oscillations. Here we analyzed the phase-locking behavior of an oscillator with a linear input term (i.e., the signal itself), but the canonical model also includes non-linear terms that govern mode locking (i.e., synchronization in integer ratios other than 1:1). For instance, the input term $x^{k}{\bar{z}}^{m-1}$ allows the oscillator, *z*, to mode-lock to the external signal, *x*, in a *k*:*m* ratio, where *k* and *m* are positive integers. With such non-linear input terms, the response of a gradient frequency network contains not only signal frequencies and natural frequencies but also non-linear responses such as harmonics, subharmonics, and combination frequencies including quadratic and cubic difference tones (Cartwright et al., 2001; Large et al., 2010), which are essential for explaining non-linearities found in the neural responses of the auditory system to acoustic signals (Large and Almonte, 2012; Lerud et al., 2014). The analysis of the canonical model with non-linear input terms, which will be given elsewhere, combines with the present analysis of linear forcing to demonstrate the full signal processing capabilities of gradient frequency neural networks.

An understanding of the relationship between auditory neurophysiology, auditory population dynamics and auditory perception remains an elusive goal, due to the intricate circuitry, the many structural levels involved, and the highly non-linear nature of the neural responses. Traditional signal processing approaches employ linear systems almost exclusively, and they approximate human perceptual capabilities only roughly. From the linear systems point of view, the basic job of the auditory system is to decompose signals into orthogonal frequency bands for subsequent pattern analysis. However, cochlear outer hair cells and auditory neurons do not decompose signals into orthogonal bands; instead, each process responds to multiple related frequencies, in a manner that is fundamentally different from linear techniques such as Fourier analysis. It appears that active networks form spatiotemporal patterns that may correspond to the perception of pitch, to the recognition of specific auditory objects, or to the induction of a beat in a musical rhythm. Significant theoretical advances will be necessary to understand signal processing, pattern formation, and plasticity in this complex and highly non-linear system. Here, we have taken the first step, by studying the responses of gradient frequency networks of non-linear oscillators forced with periodic signals. Our aim is to extend our understanding of signal processing to include more biologically realistic elements, such as oscillatory neural networks. This will enable analysis of non-linear auditory physiology from a signal processing point of view, and facilitate the design of artificial auditory networks for performing specific functions. By studying the way in which realistic neurodynamic processes respond to sounds, we hope to shed light on the remarkable capabilities of human perception that arise from non-linear processes in the auditory system.

## Author contributions

JK and EL conceived the work and wrote the manuscript. JK did the mathematical analysis.

### Conflict of interest statement

The authors declare that the research was conducted in the absence of any commercial or financial relationships that could be construed as a potential conflict of interest.
